# Post-operative delay in return of function following guided growth tension plating and use of corrective physical therapy

**DOI:** 10.1007/s11832-014-0590-3

**Published:** 2014-05-13

**Authors:** Yale A. Fillingham, Ellen Kroin, Rachel M. Frank, Brandon Erickson, Michael Hellman, Monica Kogan

**Affiliations:** Department of Orthopaedic Surgery, Rush University Medical Center, 1611 West Harrison Street, Chicago, IL 60612 USA

**Keywords:** Guided growth, Tension plating, Hemiepiphysiodesis, Angular deformity, Pediatric knee

## Abstract

**Purpose:**

Guided growth has long been used to treat growth deformities, but the Eight-Plate^®^ system has recently become more widely used by pediatric orthopaedists. Because the current literature lacks evaluation of functional status in the immediate post-operative period, we investigated functional status following use of the Eight-Plate^®^ system.

**Methods:**

We evaluated post-operative delay in return of function following treatment with the Eight-Plate^®^ system at two weeks after surgery. Fifty-one consecutive patients with a growth deformity were treated with the Eight-Plate^®^ system. Patients were comprised of 32 male and 19 female patients with an average age of 11 years (range 2–17.9 years).

**Results:**

Among study participants, 19 patients (37.3 %) had post-operative delay of function. The rate of delayed function for patients 10 years of age or younger and 11 years of age or older was respectively 11.8 and 50 % (*P* = 0.002). Six of the 19 patients were treated with four or more plates, of which five patients (83.3 %) developed delayed return of function. The rate of delayed function in patients with at least one femoral plate compared to no femoral plate was respectively 45 and 9.1 % (*P* = 0.006). Bilateral operations were associated with a 66.7 % rate of delayed function compared to 25 % with unilateral operations (*P* = 0.004). When patients with delay of function were treated with physical therapy, 12 of 13 patients (92.3 %) had complete resolution of their symptoms.

**Conclusion:**

Statistical significance demonstrated that patients at the greatest risk were 11 years of age or older, with four or more plates, with femoral plates, or with bilateral operations. Patients with delayed function were readily corrected by physical therapy.

## Introduction

Growth deformities are among the most common issues presented to pediatric orthopaedists [1]. Previously, osteotomy was the surgery of choice in children with limb length discrepancies or angular deformities of the knee, but these corrections are now achieved by newer, less traumatic techniques with devices such as Blount staples, transphyseal screws, or Eight-Plates^®^ [2–5]. Complications have been observed with the Blount staple method, including staple breakage, extrusion, changes in mechanical axis, and physis damage causing permanent closure of the growth plate [2, 6].

As a result in 2004, a novel device comprised of two screws and a two-hole plate, known as the Eight-Plate^®^ Guided Growth System (Orthofix, McKinney, TX, USA) provided advances in reversible hemiepiphyseal arrest [7]. The Eight-Plate^®^ is designed as a “tension-band,” unlike the compressive force provided by the staple, and thus allows for a lower risk of physeal fusion. The rigid screws make the device more resistant to extrusion unlike the smooth staples, and a longer moment arm should lead to a faster correction [1, 8, 9].

Standards in immediate post-operative management have been well established, such that there is no need to restrict weight-bearing, and early return to activity should be encouraged for patients. In the post-operative period, the current literature has focused on investigation into the effect of age and gender on rates of angular correction, as well as long-term complications such as re-operation following rebound growth [8, 10]. However, the current literature has primarily ignored issues with post-operative management related to delay in return of function such as pain, inability to regain full range of motion, and continued reliance on crutches.

We present a retrospective review as the first study to investigate the presence and risk factors associated with post-operative delay in return of function following the use of the Eight-Plate^®^ system. Thus, we explore the necessity to preemptively prescribe post-operative physical therapy to at-risk patients in order to more rapidly achieve full range of motion, decrease post-operative pain, and decrease the prolonged use of crutches.

## Methods

**Fig. 1:**
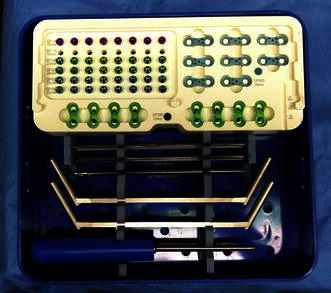
Eight-Plate^®^ guide growth system includes 12- and 16-mm plates, three sizes of cannulated screws (16, 24, and 32 mm), two sizes of solid screws (24 and 32 mm), 3.2-mm cannulated drill bit, 3.5-mm cannulated screwdriver, drill guide, and 1.6-mm K-wires

We retrospectively reviewed all 56 patients with a growth deformity who underwent guided growth using an implantable plate system, Eight-Plate^®^ (Orthofix, McKinney, TX, USA) from October 1, 2006, to July 1, 2012 (Fig. 1). The study was conducted under the approval of the institutional review board at Rush University Medical Center. The indications for surgery in this study were patients with open growth plates who had either a limb length inequality, angular deformity, or a combination of a limb length inequality and angular deformity. Contraindications to the surgery included patients with closed growth plates. The only exclusion criteria to the study were patients who had received post-operative physical therapy within the first two weeks following the operation. Physical therapy was only prescribed in the immediate post-operative two-week period at the request of the patient's parents. Based on the inclusion and exclusion criteria, this implantable system was studied in a consecutive series of 51 patients. The group comprised 32 male patients and 19 female patients. Their average age was 11.0 years (range 2.0–17.9 years). Diagnoses included limb length inequality (23), genu valgum (21), Blount’s Disease (4), genu varum (2), and flexion contracture (1). The number of plates implanted per patient included one (22), two (22), three (1), four (4), five (1), and six (1) (Table 1). All plates were implanted on the lower extremity that included distal femur only (23), distal femur and proximal tibia (17), and proximal tibia only (11) (Table 2). Among the initial 51 patients to fulfill the study criteria, a subset of 13 patients received physical therapy after more than two weeks post-operatively to help correct the delayed return of function. Delay in post-operative return of function was defined as continued pain, use of crutches, or lack of full range of motion at the initial two-week post-operative visit.

**Table 1:** Number of plates per patient

Number of plates	Patients
One plate	22
Two plates	22
Three plates	1
Four plates	4
Five plates	1
Six plates	1

**Table 2:** Number of patients based on location of plate(s)

Location of plate(s)	Patients
Distal femur only	23
Distal femur and proximal tibia	17
Proximal tibia only	11
Total	51

**Fig. 2:**
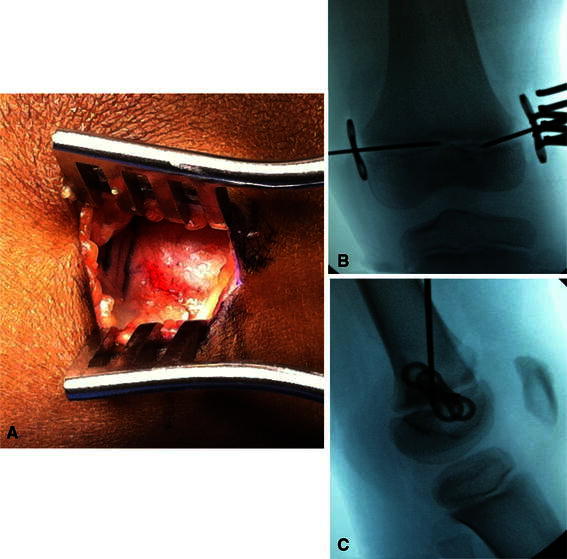
**a** Dissection was done with care to avoid violating the periosteum. **b** Intra-operative fluoroscopic image showing identification of the physis (AP). **c** Intra-operative fluoroscopic image showing identification of the physis (Lateral)

**Fig. 3:**
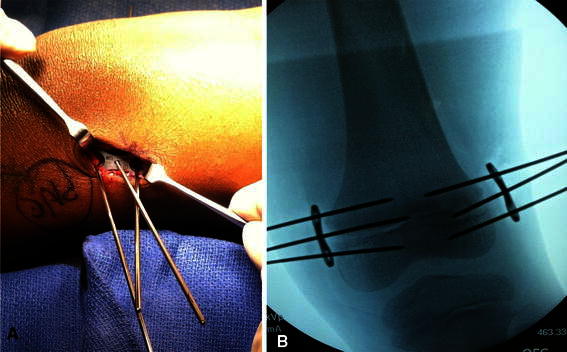
**a** Demonstrates placement of plates being held in place by 1.6-mm K-wires. **b** Intra-operative fluoroscopic image of Eight-Plate^®^ held in place with 1.6-mm K-wires through the epiphysis and metaphysis

**Fig. 4:**
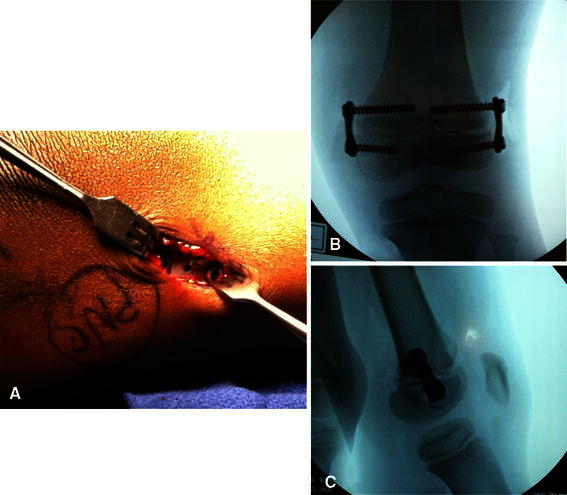
**a** Final placement of plates visualized. **b** Final intra-operative fluoroscopic image verifying plate and screw placement (AP). **c** Final intra-operative fluoroscopic image verifying plate and screw placement (Lateral)

All patients were treated by one surgeon (MK). The procedure was performed following the manufacturer’s operative technique. Under the guidance of the fluoroscopic image intensifier the physis was identified, followed by dissection down to the periosteum with care taken not to violate the periosteum (Fig. 2a–c). Approaching the distal femur from the lateral side was done by incising through the iliotibial band to reach the periosteum. The medial side of the distal femur was approached by dissection posterior to the vastus medialis to reach the periosteum. The appropriate sized Eight-Plate^®^ was selected and held in place with guide wires in both the epiphyseal and metaphyseal plate holes (Fig. 3a, b). Once the position was verified with the fluoroscope, both holes were drilled, and cannulated self-tapping screws were used to secure the location of the plate. After placement of both screws the final position of the plate and screws were verified, taking note to ensure the screws did not cross the physis or penetrate the articular cartilage (Fig. 4a–c).

Post-operatively, patients were provided crutches strictly for comfort measures, but were instructed to be full weight-bearing. They were not provided formal home exercises, but they were given instructions to encourage early range of motion as well as to return to normal activity as tolerated on the effected lower extremity, and to discontinue the crutches as tolerated.

All procedures were performed on an outpatient basis because post-operatively the patients’ pain was controlled with oral pain medications and did not have any further indications requiring an inpatient setting. After discharge from the hospital, the patients were followed in the clinic at two weeks and then intervals of three months to document progression and identify timing of hardware removal. The follow-up intervals were altered at the surgeon’s discretion owing to variable growth rates, variable levels of correction required, and encountered delays in return of function. Each routine follow-up in the clinic included assessment of strength, range of motion, functional status, and radiographic investigation to evaluate for correction of growth deformity and hardware position.

We retrospectively reviewed the defined patient population that included verification of the diagnosis, operative date, location of plates implanted, number of plates implanted, and pre-operative functional status. Patients were investigated for the presence of post-operative delay in return of function at the initial two-week follow-up appointment, which was defined as presence of pain, use of crutches, or lack of full range of motion. The presence of pain was classified as any level of pain that prevented the patients from returning to their pre-operative level of function. When patients were noted to have functional pain, use of crutches, or lacked full range of motion at the two-week post-operative visit, they were label as having delayed return of function. Any patients who received physical therapy due to delayed return of function were tracked for resolution of symptoms during their subsequent follow-up appointments.

## Results

**Fig. 5:**
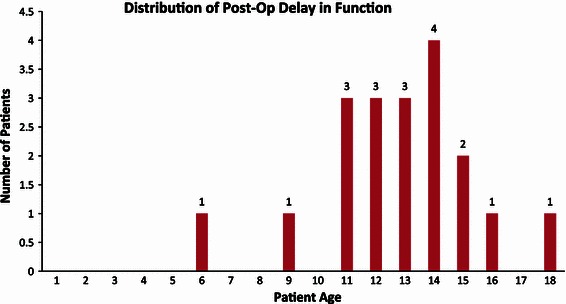
Graphic illustration demonstrates the distinctive distribution of post-operative delay in function among patients 11 years of age and older

The occurrence of post-operative delay in return of function at two weeks was present in 19 of 51 patients (37.3 %) with complaints of lacking full range of motion (14; 73.7 %), use of crutches (2; 10.5 %), presence of pain (1; 5.3 %), presence of pain and lacking full range of motion (1; 5.3 %), and presence of pain and use of crutches (1; 5.3 %). The two patients presenting with more than one complaint included a 13-year-old male and 18-year-old male both treated for genu valgum with the placement of two plates including the femur. Both patients underwent delayed physical therapy and experienced resolution of their symptoms. Among the 19 patients to experience post-operative delay in return of function, 17 patients (89.5 %) were 11 years of age or older (Fig. 5). The rate of delayed return of function was analyzed among the study population of the 34 patients who were 11 years of age or older and of the 17 patients who were 10 years of age or younger. Seventeen of those 34 patients (50.0 %) who were 11 years of age or older were found to have post-operative delay in return of function. Meanwhile, only two of the 17 patients (11.8 %) who were 10 years or younger experienced a delay in return of function. The resulting rate of delayed return of function for the two age groups was respectively 50 and 11.8 % (*P* = 0.002).

Within the 51 patients, 45 patients had less than four plates placed, while six patients had four or more plates placed. Five of the six patients (83.3 %) treated with four or more plates developed delayed return of function, while only 14 of the 45 patients (31.1 %) with less than four plates demonstrated a delay in function (*P* = 0.02). The distribution in the location of the plates among the 19 patients to have delayed return of function included the distal femur and proximal tibia (10), distal femur only (8), and proximal tibia only (1). As a result, 18 of the 19 patients (94.7 %) with post-operative delay in return of function had at least one femoral plate. The study included 40 patients with at least one femoral plate and 11 patients with no femoral plates. The rate of delayed return of function between the patients with at least one femoral plate and no femoral plates was respectively 45 and 9.1 % (*P* = 0.006). Among the study population, 15 patients underwent bilateral placement of the plates at the time of operation. Ten of the 15 patients (66.7 %) with a bilateral operation developed delayed return of function, but only nine of the 36 patients (25 %) with a unilateral operation had a delay in function (*P* = 0.004).

All of the 19 patients who had a post-operative delay in return of function at two weeks were given the option to start physical therapy, but only 13 patients completed physical therapy. Twelve of the 13 patients (92.3 %) who underwent physical therapy were noted to have complete resolution of their symptoms during future follow-up appointments. The one patient who did not have resolution following physical therapy was a 9.5-year-old female treated for limb length inequality with the placement of four plates who continued to lack full range of motion in her left knee.

## Discussion

The basic principles of harnessing the ability of growing bone to correct growth deformities have been well-known to the field of bone and joint surgery for centuries [1]. The vast majority of the recent advancement in the treatment of growth deformities has been in the use of temporary hemiepiphysiodesis with the Eight-Plate^®^ system due to its high rates of successful correction and minimal complications [7, 10–17]. While the current literature has primarily focused on measuring the success of treatment and long-term complications related to hardware failure in Blount’s Disease, it lacks evaluation of functional status in the immediate post-operative period [7, 10–18]. We have performed a retrospective study to identify patients at risk for post-operative delay in return of function following treatment with the Eight-Plate^®^ system. We believe it is important to address these issues to limit the significant social implications for the patients as well as for the caregiver.

We acknowledge several limitations to our study. First, it is a relatively small study with no emphasis placed on long-term follow-up. Given that the purpose of the study was on the immediate post-operative period, we believe the lack of long-term follow-up does little to detract from the conclusions of the study. Second, the patient population is heterogeneous by including patients with multiple diagnoses and varying degrees of correction. As a result of the small number of subjects and heterogeneity in the study, comparison of diagnosis and degree of correction with post-operative function was unreliable. Third, the use of pain as criteria for defining delayed return of function was measured subjectively as any pain preventing the patients from returning to their pre-operative level of function. We did not use the visual analogue scale to quantify their pain level. As a result, we are unable to more accurately comment on the level of the patients’ pain and understand the severity of the pain in relation to their functional deficit. Lastly, we performed a retrospective study, and thus carry significant limitations inherent to the study design.

General consensus in the literature has been to encourage early weight-bearing, range of motion, and return to activity following the placement of the Eight-Plate^®^ system [7, 10, 11, 15]. However, few studies have discussed the immediate post-operative course and timing of return to normal activity for these patients. When Burghardt et al. [7] studied the effectiveness of the Eight-Plate^®^ in the correction of angular deformities in children younger than 5 years of age, physical therapy was prescribed if the patient was not bending the knee at the first follow-up visit (7–14 days post-operative). Although Burghardt et al. [7, 15] and Stevens [15] mentioned experiencing post-operative delay in return of function in each of their studies, the frequency of occurrence and the success of delayed post-operative physical therapy was unreported. In the observational study on the effectiveness of the Eight-Plate^®^ in the correction of angular deformities in 25 children, Ballal et al. [10] mentions that full weight-bearing was usually achieved in the second post-operative week. However, the post-operative course differed from our study because Ballal et al. [10] kept the patients overnight, discharged them as partial weight-bearing on crutches with a compressive dressing removed after 3–4 days, and then encouraged knee motion.

In our study, only 19 of 51 patients were reported to have a delayed return of function, but a significant portion of those patients were 11 years of age and older. When patients 11 years of age and older are isolated from the rest of the study population, the patients with delay in function accounts for 17 of 34 patients (50 %). The rate of delayed return of function for older patients was significantly greater than the rate of 11.8 % among patients 10 years of age or younger. The results demonstrate a statistically significant rate of delayed return of function between the two age groups.

In addition to patient age, we demonstrate a statistically significant correlation in post-operative delay in function with the number of plates, location of plates, and patients undergoing a bilateral operation. Based on the results of our study, 83.3 % of patients with four or more plates versus 31.1 % of patients with less than four plates experienced a delay in return of function. We have also demonstrated with statistical significance that the patients undergoing a bilateral operation experienced a delay in function more frequently than patients with a unilateral operation. The rates of delayed return of function for the two groups were respectively 66.7 and 25 %. Burghardt et al. [7] noted that patients with placement of femoral plates were more likely to require physical therapy for lacking knee range of motion at the first follow-up visit. We validated the findings of Burghardt et al., with 18 of 19 patients experiencing post-operative delay in function when there was the placement of at least one distal femur plate. Additionally, we illustrate a statistically significant difference between the rate of delayed return of function in patients with at least one femoral plate (45 %) compared to patients with no femoral plates (9.1 %). Despite the limitation presented by the small number of patients in the conclusion regarding number and location of plates, we are able to validate these conclusions from a prior study.

Although analysis exhibited with statistical significance a higher rate of delayed post-operative return of function for patients 11 years of age and older, we believe the finding is an artifact of other factors not explicitly related to an underlying physiological process unique to older patients. When older patients present with conditions indicated for treatment with guided growth, a more aggressive treatment plan is required due to the limited period of time to obtain the desired correction. The result was older patients more frequently requiring a greater number of plates, bilateral operations, and use of femoral plates, which was consistent with the patients treated in our study. The group of patients 11 years of age and older in our study consistently had a higher incidence of using more than one plate, requiring bilateral operations, or using femoral plates. We have identified in the study an increased rate of delayed post-operative return of function with the use of a greater number of plates, bilateral operations, and use of femoral plates, which resulted in the perceived bias of the patient’s age on the risk of delayed return of function.

We believe the approach to the distal femur causes femoral plates to be more associated with functional pain, use of crutches, or impaired range of motion. Dissection of the distal femur for placement of the plates involves the iliotibial band or vastus medialis, which are both actively involved in the biomechanics of the knee. In addition, accurate insertion of a plate on the lateral aspect of the distal femur commonly requires violation of the joint capsule. Iatrogenic injury to any of these structures, and particularly the joint capsule, would contribute to the symptoms of post-operative delay in return of function. As for the etiology of the delayed function with the use of a greater number of plates and bilateral operations, it is intuitive that both will lead to more soft tissue manipulation, causing increased post-operative pain and range of motion impairment.

When previous studies discussed the use of physical therapy for decreased range of motion or lack of full weight-bearing, the results of initiating physical therapy for these patients were unreported [7, 15]. We found that the use of delayed physical therapy led to a return in function for 92.3 % of patients. Although delayed physical therapy does not help patients in the immediate post-operative period, it still provided improved outcomes for patients.

As the Eight-Plate^®^ guided growth system continues to become more widely used in the treatment of growth deformities, we need to be able to identify the patients at risk for delayed return in function during the immediate post-operative period. We have presented the first study focused on identifying patients at risk of post-operative delay in return of function, which demonstrates patients 11 years of age and older, patients with four or more plates, patients with femoral plates, or patients undergoing a bilateral operation to be most at risk. The majority of the patients with post-operative delay in return of function were unable to return to school because of issues with the use of crutches in school or pain control. Although we demonstrated that delayed physical therapy leads to resolution of the patient’s symptoms, we recommend initiating physical therapy immediately for at-risk patients. Despite our study demonstrating the presence of post-operative delay in the return of function for specific patients, we suggest the future need for a well-designed comparative study to validate the effectiveness of immediate post-operative physical therapy in the prevention of delayed return of function.
